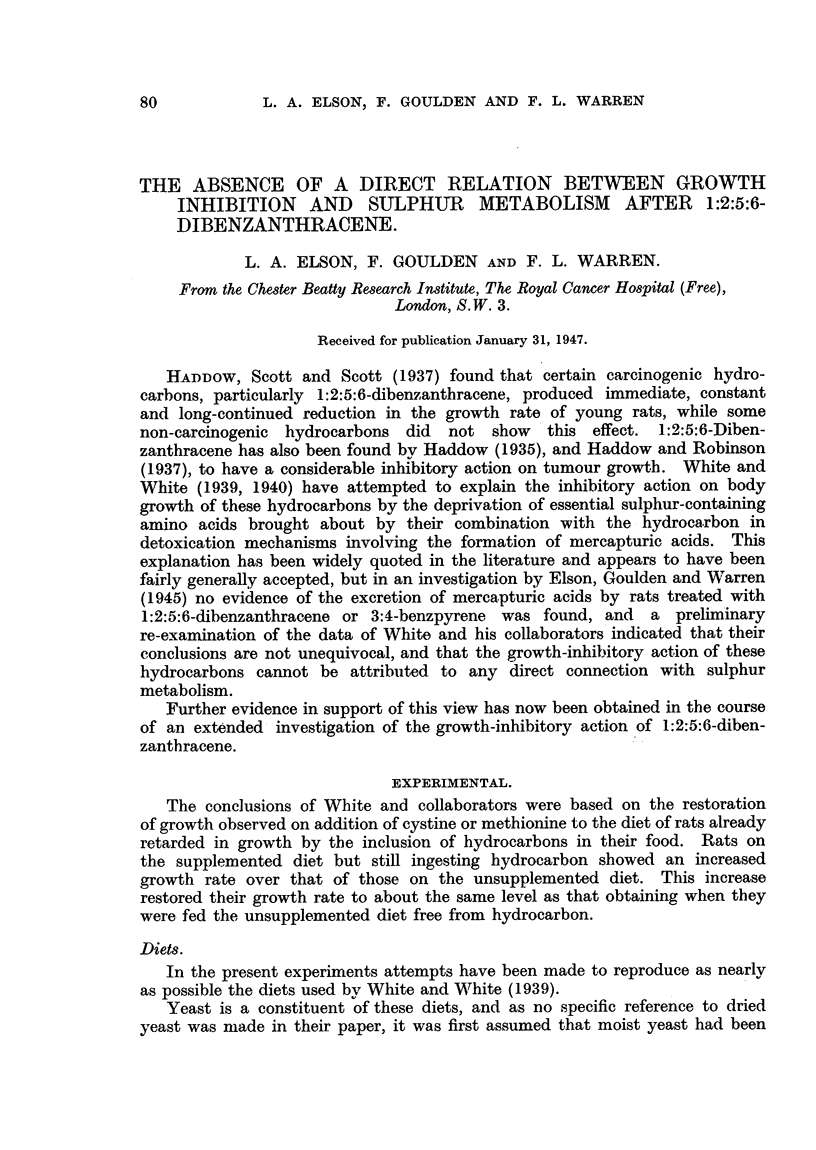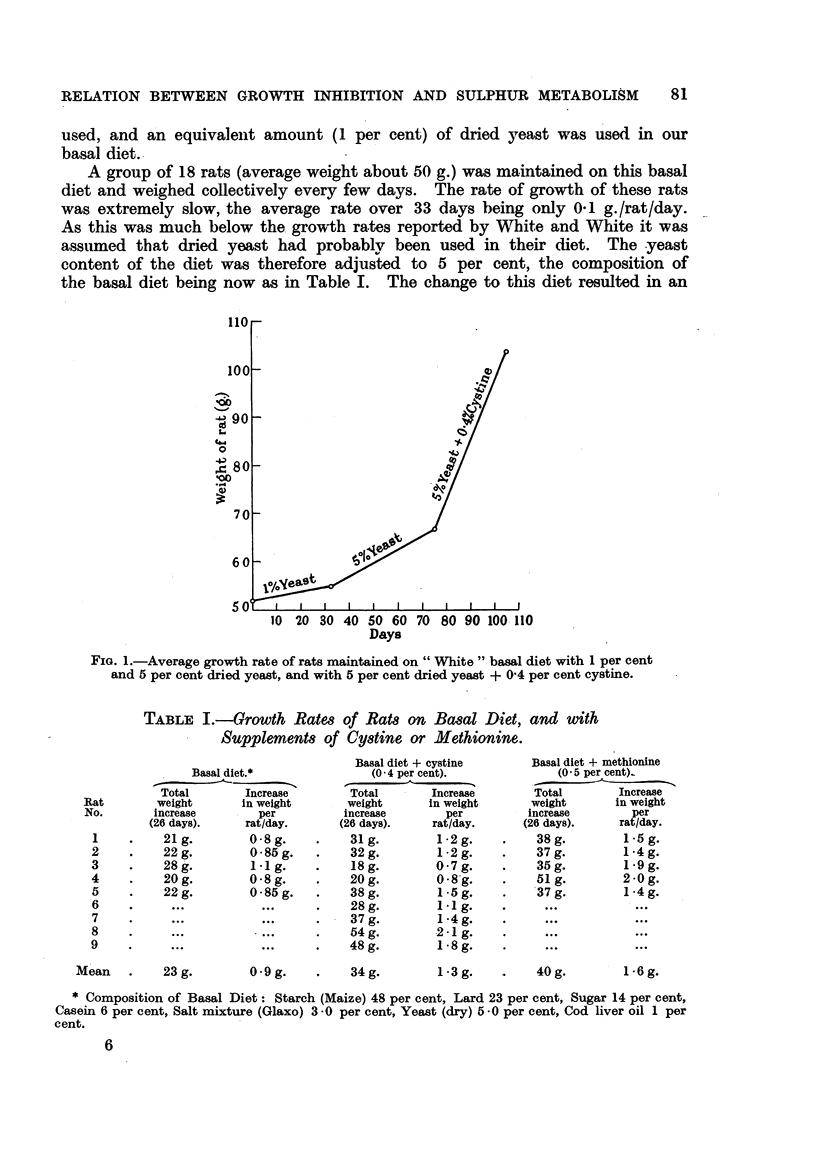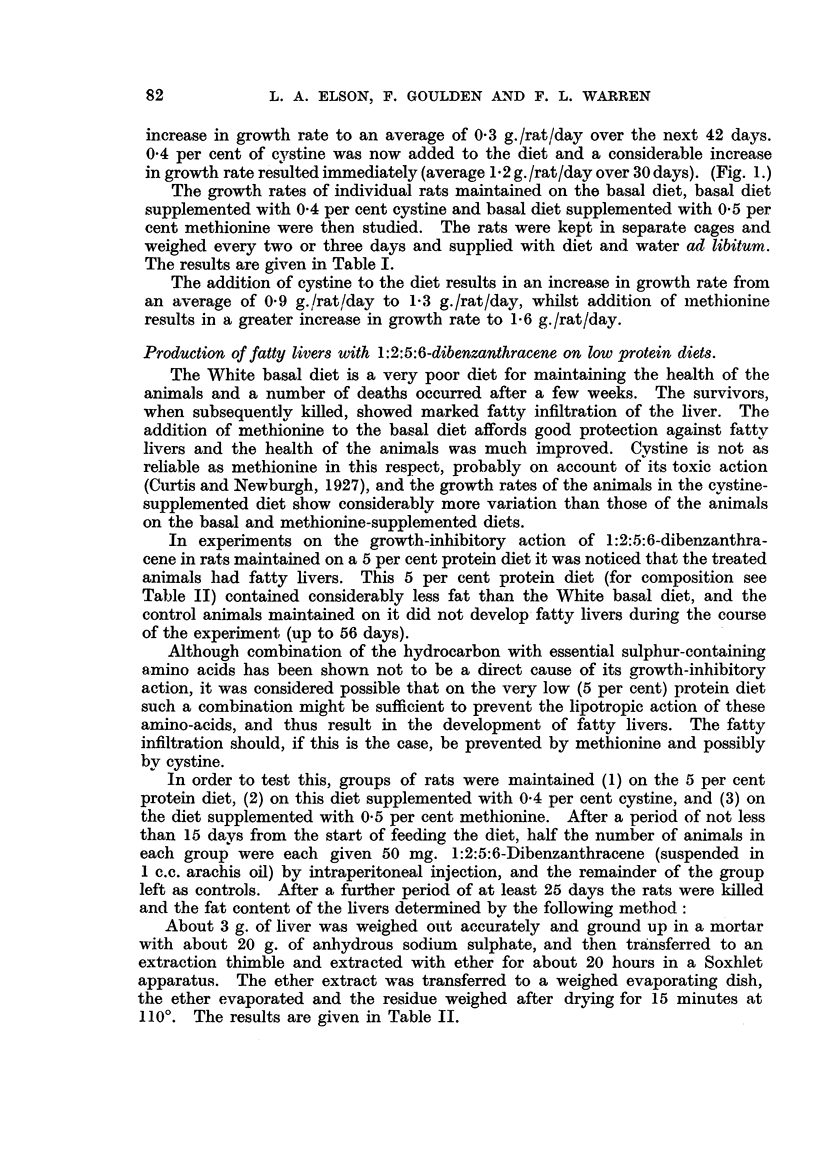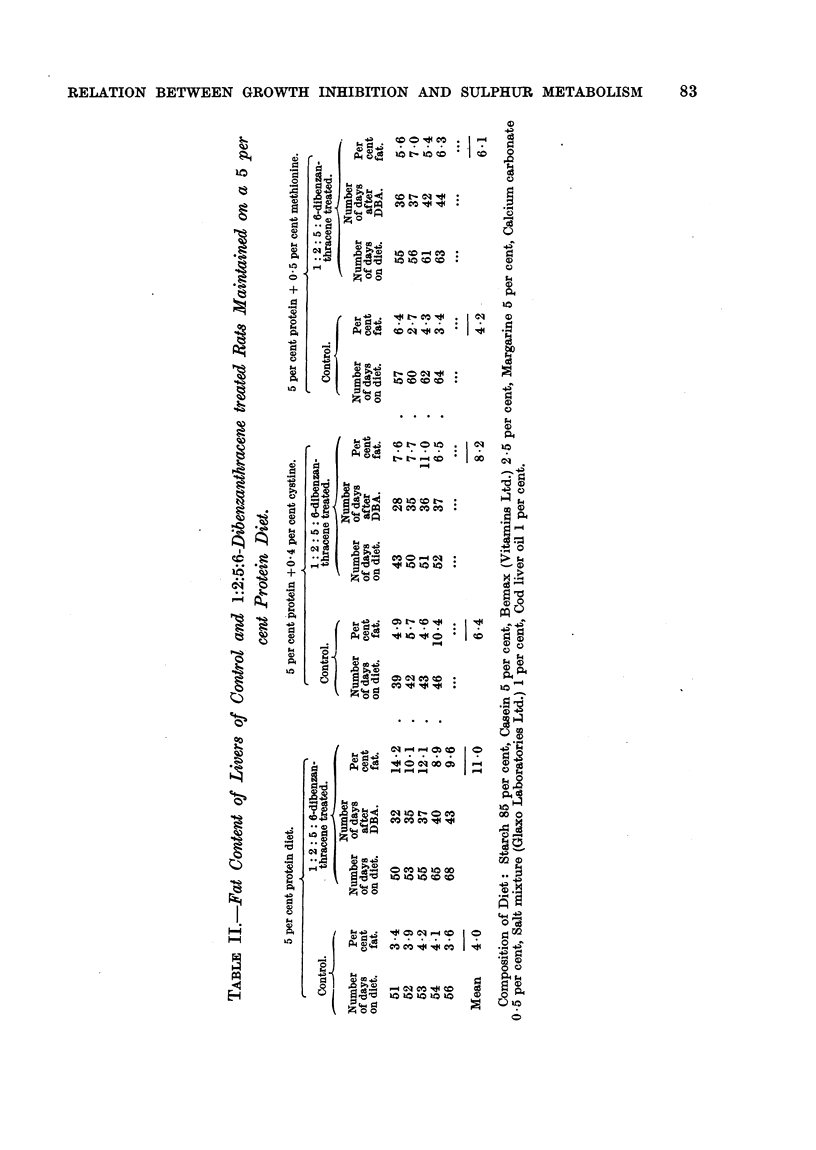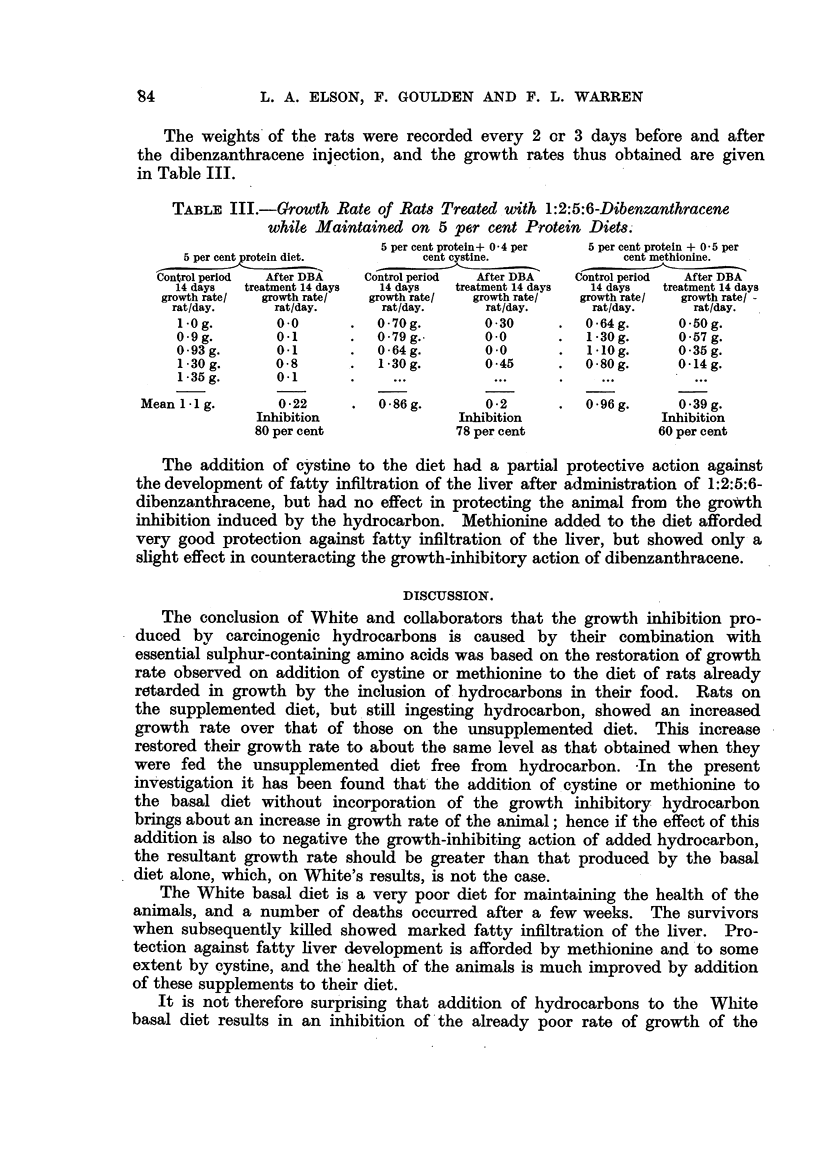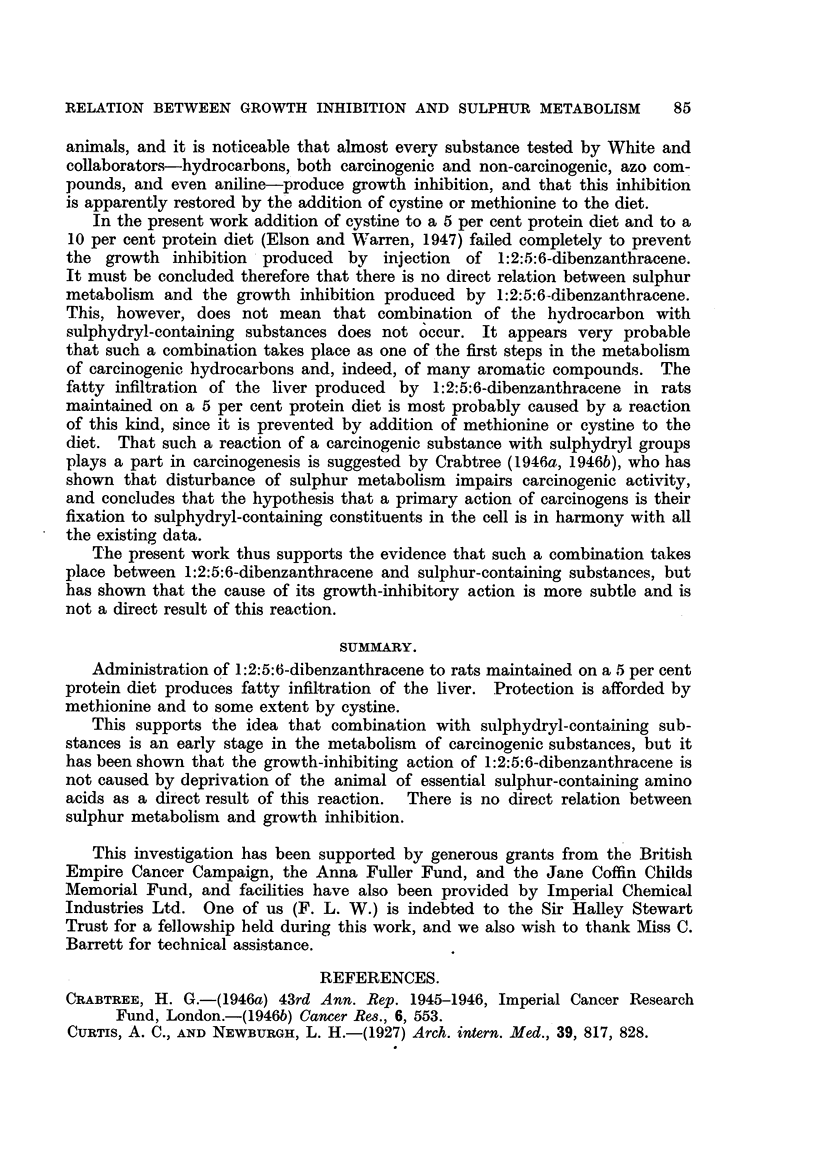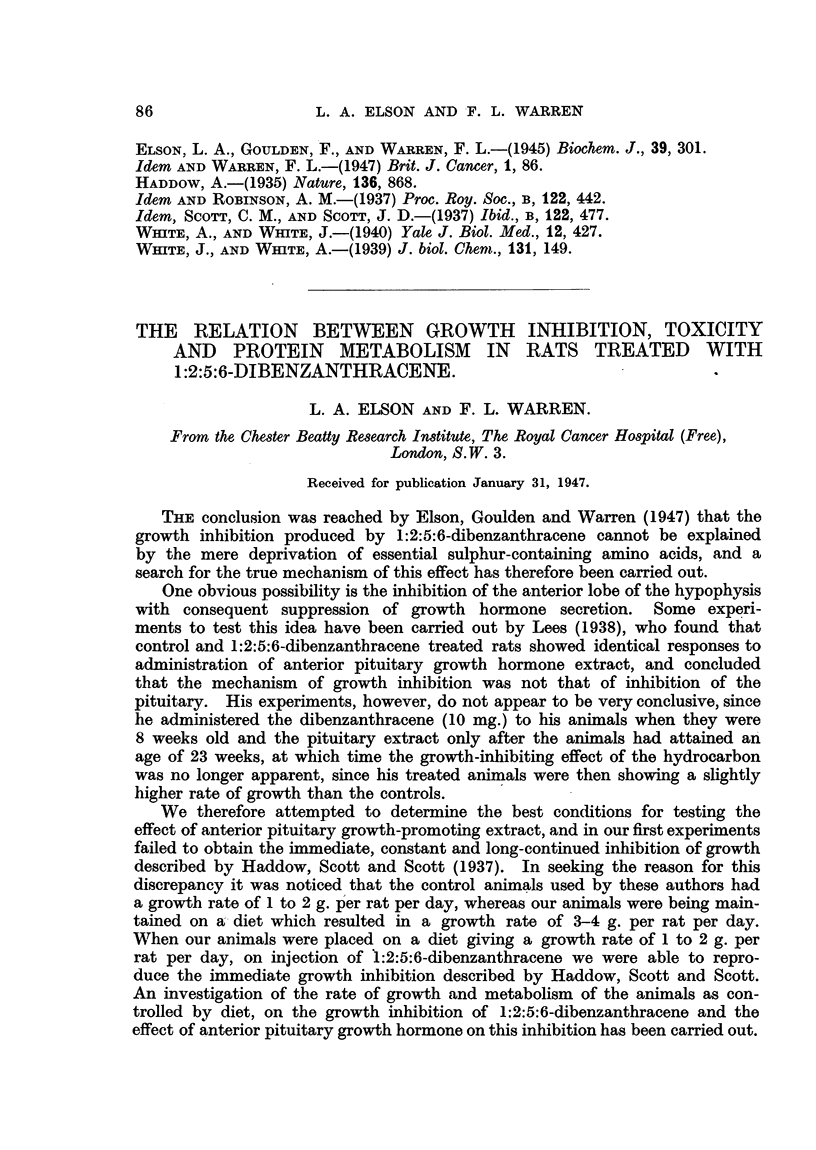# The Absence of a Direct Relation between Growth Inhibition and Sulphur Metabolism After 1:2:5:6-Dibenzanthracene

**DOI:** 10.1038/bjc.1947.10

**Published:** 1947-03

**Authors:** L. A. Elson, F. Goulden, F. L. Warren


### THE ABSENCE OF A DIRECT RELATION BETWEEN GROWTH INHIBITION AND SULPHUR METABOLISM AFTER 1:2:5:6-DIBENZANTHRACENE.

L. A. ELSON, F. GOULDEN AND F. L. WARREN.

*From the Chester Beatty Research Institute, The Royal Cancer Hospital (Free), London, S.W. 3.*

Received for publication January 31, 1947.

HADDOW, Scott and Scott (1937) found that certain carcinogenic hydrocarbons, particularly 1:2:5:6-dibenzanthracene, produced immediate, constant and long-continued reduction in the growth rate of young rats, while some non-carcinogenic hydrocarbons did not show this effect. 1:2:5:6-Dibenzanthracene has also been found by Haddow (1935), and Haddow and Robinson (1937), to have a considerable inhibitory action on tumour growth. White and White (1939, 1940) have attempted to explain the inhibitory action on body growth of these hydrocarbons by the deprivation of essential sulphur-containing amino acids brought about by their combination with the hydrocarbon in detoxication mechanisms involving the formation of mercapturic acids. This explanation has been widely quoted in the literature and appears to have been fairly generally accepted, but in an investigation by Elson, Goulden and Warren (1945) no evidence of the excretion of mercapturic acids by rats treated with 1:2:5:6-dibenzanthracene or 3:4-benzpyrene was found, and a preliminary re-examination of the data of White and his collaborators indicated that their conclusions are not unequivocal, and that the growth-inhibitory action of these hydrocarbons cannot be attributed to any direct connection with sulphur metabolism.

Further evidence in support of this view has now been obtained in the course of an extended investigation of the growth-inhibitory action of 1:2:5:6-dibenzanthracene.

### EXPERIMENTAL.

The conclusions of White and collaborators were based on the restoration of growth observed on addition of cystine or methionine to the diet of rats already retarded in growth by the inclusion of hydrocarbons in their food. Rats on the supplemented diet but still ingesting hydrocarbon showed an increased growth rate over that of those on the unsupplemented diet. This increase restored their growth rate to about the same level as that obtaining when they were fed the unsupplemented diet free from hydrocarbon.

### *Diets.*

In the present experiments attempts have been made to reproduce as nearly as possible the diets used by White and White (1939).

Yeast is a constituent of these diets, and as no specific reference to dried yeast was made in their paper, it was first assumed that moist yeast had been used, and an equivalent amount (1 per cent) of dried yeast was used in our basal diet.

A group of 18 rats (average weight about 50 g.) was maintained on this basal diet and weighed collectively every few days. The rate of growth of these rats was extremely slow, the average rate over 33 days being only 0·1 g./rat/day. As this was much below the growth rates reported by White and White it was assumed that dried yeast had probably been used in their diet. The yeast content of the diet was therefore adjusted to 5 per cent, the composition of the basal diet being now as in Table I. The change to this diet resulted in an

FIG. 1.—Average growth rate of rats maintained on " White " basal diet with 1 per cent and 5 per cent dried yeast, and with 5 per cent dried yeast + 0·4 per cent cystine.

TABLE I.—*Growth Rates of Rats on Basal Diet, and with Supplements of Cystine or Methionine.*

| Rat No. | Basal diet.* | | Basal diet + cystine (0·4 per cent). | | Basal diet + methionine (0·5 per cent). | |
|---|---|---|---|---|---|---|
| | Total weight increase (26 days). | Increase in weight per rat/day. | Total weight increase (26 days). | Increase in weight per rat/day. | Total weight increase (26 days). | Increase in weight per rat/day. |
| 1 | 21 g. | 0·8 g. | 31 g. | 1·2 g. | 38 g. | 1·5 g. |
| 2 | 22 g. | 0·85 g. | 32 g. | 1·2 g. | 37 g. | 1·4 g. |
| 3 | 28 g. | 1·1 g. | 18 g. | 0·7 g. | 35 g. | 1·9 g. |
| 4 | 20 g. | 0·8 g. | 20 g. | 0·8 g. | 51 g. | 2·0 g. |
| 5 | 22 g. | 0·85 g. | 38 g. | 1·5 g. | 37 g. | 1·4 g. |
| 6 | ... | ... | 28 g. | 1·1 g. | ... | ... |
| 7 | ... | ... | 37 g. | 1·4 g. | ... | ... |
| 8 | ... | ... | 54 g. | 2·1 g. | ... | ... |
| 9 | ... | ... | 48 g. | 1·8 g. | ... | ... |
| Mean | 23 g. | 0·9 g. | 34 g. | 1·3 g. | 40 g. | 1·6 g. |

* Composition of Basal Diet: Starch (Maize) 48 per cent, Lard 23 per cent, Sugar 14 per cent, Casein 6 per cent, Salt mixture (Glaxo) 3·0 per cent, Yeast (dry) 5·0 per cent, Cod liver oil 1 per cent.

increase in growth rate to an average of 0·3 g./rat/day over the next 42 days. 0·4 per cent of cystine was now added to the diet and a considerable increase in growth rate resulted immediately (average 1·2 g./rat/day over 30 days). (Fig. 1.)

The growth rates of individual rats maintained on the basal diet, basal diet supplemented with 0·4 per cent cystine and basal diet supplemented with 0·5 per cent methionine were then studied. The rats were kept in separate cages and weighed every two or three days and supplied with diet and water *ad libitum*. The results are given in Table I.

The addition of cystine to the diet results in an increase in growth rate from an average of 0·9 g./rat/day to 1·3 g./rat/day, whilst addition of methionine results in a greater increase in growth rate to 1·6 g./rat/day.

*Production of fatty livers with* 1:2:5:6-*dibenzanthracene on low protein diets.*

The White basal diet is a very poor diet for maintaining the health of the animals and a number of deaths occurred after a few weeks. The survivors, when subsequently killed, showed marked fatty infiltration of the liver. The addition of methionine to the basal diet affords good protection against fatty livers and the health of the animals was much improved. Cystine is not as reliable as methionine in this respect, probably on account of its toxic action (Curtis and Newburgh, 1927), and the growth rates of the animals in the cystine-supplemented diet show considerably more variation than those of the animals on the basal and methionine-supplemented diets.

In experiments on the growth-inhibitory action of 1:2:5:6-dibenzanthracene in rats maintained on a 5 per cent protein diet it was noticed that the treated animals had fatty livers. This 5 per cent protein diet (for composition see Table II) contained considerably less fat than the White basal diet, and the control animals maintained on it did not develop fatty livers during the course of the experiment (up to 56 days).

Although combination of the hydrocarbon with essential sulphur-containing amino acids has been shown not to be a direct cause of its growth-inhibitory action, it was considered possible that on the very low (5 per cent) protein diet such a combination might be sufficient to prevent the lipotropic action of these amino-acids, and thus result in the development of fatty livers. The fatty infiltration should, if this is the case, be prevented by methionine and possibly by cystine.

In order to test this, groups of rats were maintained (1) on the 5 per cent protein diet, (2) on this diet supplemented with 0·4 per cent cystine, and (3) on the diet supplemented with 0·5 per cent methionine. After a period of not less than 15 days from the start of feeding the diet, half the number of animals in each group were each given 50 mg. 1:2:5:6-Dibenzanthracene (suspended in 1 c.c. arachis oil) by intraperitoneal injection, and the remainder of the group left as controls. After a further period of at least 25 days the rats were killed and the fat content of the livers determined by the following method :

About 3 g. of liver was weighed out accurately and ground up in a mortar with about 20 g. of anhydrous sodium sulphate, and then transferred to an extraction thimble and extracted with ether for about 20 hours in a Soxhlet apparatus. The ether extract was transferred to a weighed evaporating dish, the ether evaporated and the residue weighed after drying for 15 minutes at 110°. The results are given in Table II.

The weights of the rats were recorded every 2 or 3 days before and after the dibenzanthracene injection, and the growth rates thus obtained are given in Table III.

TABLE III.—*Growth Rate of Rats Treated with* 1:2:5:6-*Dibenzanthracene while Maintained on* 5 *per cent Protein Diets.*

| 5 per cent protein diet. | | 5 per cent protein + 0·4 per cent cystine. | | 5 per cent protein + 0·5 per cent methionine. | |
|---|---|---|---|---|---|
| Control period 14 days growth rate/ rat/day. | After DBA treatment 14 days growth rate/ rat/day. | Control period 14 days growth rate/ rat/day. | After DBA treatment 14 days growth rate/ rat/day. | Control period 14 days growth rate/ rat/day. | After DBA treatment 14 days growth rate/ rat/day. |
| 1·0 g. | 0·0 | 0·70 g. | 0·30 | 0·64 g. | 0·50 g. |
| 0·9 g. | 0·1 | 0·79 g. | 0·0 | 1·30 g. | 0·57 g. |
| 0·93 g. | 0·1 | 0·64 g. | 0·0 | 1·10 g. | 0·35 g. |
| 1·30 g. | 0·8 | 1·30 g. | 0·45 | 0·80 g. | 0·14 g. |
| 1·35 g. | 0·1 | ... | ... | ... | ... |
| Mean 1·1 g. | 0·22 Inhibition 80 per cent | 0·86 g. | 0·2 Inhibition 78 per cent | 0·96 g. | 0·39 g. Inhibition 60 per cent |

The addition of cystine to the diet had a partial protective action against the development of fatty infiltration of the liver after administration of 1:2:5:6-dibenzanthracene, but had no effect in protecting the animal from the growth inhibition induced by the hydrocarbon. Methionine added to the diet afforded very good protection against fatty infiltration of the liver, but showed only a slight effect in counteracting the growth-inhibitory action of dibenzanthracene.

### DISCUSSION.

The conclusion of White and collaborators that the growth inhibition produced by carcinogenic hydrocarbons is caused by their combination with essential sulphur-containing amino acids was based on the restoration of growth rate observed on addition of cystine or methionine to the diet of rats already retarded in growth by the inclusion of hydrocarbons in their food. Rats on the supplemented diet, but still ingesting hydrocarbon, showed an increased growth rate over that of those on the unsupplemented diet. This increase restored their growth rate to about the same level as that obtained when they were fed the unsupplemented diet free from hydrocarbon. In the present investigation it has been found that the addition of cystine or methionine to the basal diet without incorporation of the growth inhibitory hydrocarbon brings about an increase in growth rate of the animal; hence if the effect of this addition is also to negative the growth-inhibiting action of added hydrocarbon, the resultant growth rate should be greater than that produced by the basal diet alone, which, on White's results, is not the case.

The White basal diet is a very poor diet for maintaining the health of the animals, and a number of deaths occurred after a few weeks. The survivors when subsequently killed showed marked fatty infiltration of the liver. Protection against fatty liver development is afforded by methionine and to some extent by cystine, and the health of the animals is much improved by addition of these supplements to their diet.

It is not therefore surprising that addition of hydrocarbons to the White basal diet results in an inhibition of the already poor rate of growth of the animals, and it is noticeable that almost every substance tested by White and collaborators—hydrocarbons, both carcinogenic and non-carcinogenic, azo compounds, and even aniline—produce growth inhibition, and that this inhibition is apparently restored by the addition of cystine or methionine to the diet.

In the present work addition of cystine to a 5 per cent protein diet and to a 10 per cent protein diet (Elson and Warren, 1947) failed completely to prevent the growth inhibition produced by injection of 1:2:5:6-dibenzanthracene. It must be concluded therefore that there is no direct relation between sulphur metabolism and the growth inhibition produced by 1:2:5:6-dibenzanthracene. This, however, does not mean that combination of the hydrocarbon with sulphydryl-containing substances does not occur. It appears very probable that such a combination takes place as one of the first steps in the metabolism of carcinogenic hydrocarbons and, indeed, of many aromatic compounds. The fatty infiltration of the liver produced by 1:2:5:6-dibenzanthracene in rats maintained on a 5 per cent protein diet is most probably caused by a reaction of this kind, since it is prevented by addition of methionine or cystine to the diet. That such a reaction of a carcinogenic substance with sulphydryl groups plays a part in carcinogenesis is suggested by Crabtree (1946*a*, 1946*b*), who has shown that disturbance of sulphur metabolism impairs carcinogenic activity, and concludes that the hypothesis that a primary action of carcinogens is their fixation to sulphydryl-containing constituents in the cell is in harmony with all the existing data.

The present work thus supports the evidence that such a combination takes place between 1:2:5:6-dibenzanthracene and sulphur-containing substances, but has shown that the cause of its growth-inhibitory action is more subtle and is not a direct result of this reaction.

### SUMMARY.

Administration of 1:2:5:6-dibenzanthracene to rats maintained on a 5 per cent protein diet produces fatty infiltration of the liver. Protection is afforded by methionine and to some extent by cystine.

This supports the idea that combination with sulphydryl-containing substances is an early stage in the metabolism of carcinogenic substances, but it has been shown that the growth-inhibiting action of 1:2:5:6-dibenzanthracene is not caused by deprivation of the animal of essential sulphur-containing amino acids as a direct result of this reaction. There is no direct relation between sulphur metabolism and growth inhibition.

This investigation has been supported by generous grants from the British Empire Cancer Campaign, the Anna Fuller Fund, and the Jane Coffin Childs Memorial Fund, and facilities have also been provided by Imperial Chemical Industries Ltd. One of us (F. L. W.) is indebted to the Sir Halley Stewart Trust for a fellowship held during this work, and we also wish to thank Miss C. Barrett for technical assistance.

### REFERENCES.

CRABTREE, H. G.—(1946*a*) *43rd Ann. Rep.* 1945–1946, Imperial Cancer Research Fund, London.—(1946*b*) *Cancer Res.*, **6**, 553.

CURTIS, A. C., AND NEWBURGH, L. H.—(1927) *Arch. intern. Med.*, **39**, 817, 828.

ELSON, L. A., GOULDEN, F., AND WARREN, F. L.—(1945) *Biochem. J.*, **39**, 301.
*Idem* AND WARREN, F. L.—(1947) *Brit. J. Cancer*, **1**, 86.
HADDOW, A.—(1935) *Nature*, **136**, 868.
*Idem* AND ROBINSON, A. M.—(1937) *Proc. Roy. Soc.*, B, **122**, 442.
*Idem*, SCOTT, C. M., AND SCOTT, J. D.—(1937) *Ibid.*, B, **122**, 477.
WHITE, A., AND WHITE, J.—(1940) *Yale J. Biol. Med.*, **12**, 427.
WHITE, J., AND WHITE, A.—(1939) *J. biol. Chem.*, **131**, 149.

---

### THE RELATION BETWEEN GROWTH INHIBITION, TOXICITY AND PROTEIN METABOLISM IN RATS TREATED WITH 1:2:5:6-DIBENZANTHRACENE.

L. A. ELSON AND F. L. WARREN.